# Live birth rate comparison of three controlled ovarian stimulation protocols for in vitro fertilization-embryo transfer in patients with diminished ovarian reserve after endometrioma cystectomy: a retrospective study

**DOI:** 10.1186/s13048-020-00622-x

**Published:** 2020-02-29

**Authors:** Feiyan Zhao, Yonglian Lan, Tong Chen, Zhimin Xin, Yu Liang, Ying Li, Shuyu Wang, Jun Zhang, Xiaokui Yang

**Affiliations:** Department of Human Reproductive Medicine, Beijing Obstetrics and Gynecology Hospital, Capital Medical University, 251 Yao jia yuan Road, Chaoyang District, Beijing, 100026 China

**Keywords:** In vitro fertilization-embryo transfer, Live birth, Cystectomy, Diminished ovarian reserve, Endometrioma

## Abstract

**Background:**

Women with endometriosis and previous cystectomy may respond less well to gonadotropin stimulation, which results in fewer oocytes retrieved and poor pregnancy outcomes. Choosing an appropriate protocol for such populations is essential. This study involved an analysis of the effect of different controlled ovarian stimulation (COS) protocols on the clinical outcomes of in vitro fertilization-embryo transfer (IVF-ET) in women with diminished ovarian reserve (DOR) who underwent ovarian endometrioma cystectomy.

**Methods:**

A total of 342 patients that underwent IVF-ET treatment at the Beijing Obstetrics and Gynecology Hospital from January 1, 2013 to April 30, 2018 were included in this retrospective study. The patients were distributed into three groups according to the COS protocols, namely prolonged GnRH-agonist (Group A, *n* = 113), GnRH-antagonist (Group B, *n* = 121), and long GnRH-agonist (Group C, *n* = 108). The clinical and laboratory parameters of the three protocols were analyzed and a logistic regression of clinical pregnancy and live births was conducted.

**Results:**

There were no significant differences in the age, infertility duration, basic follicle stimulation hormone (FSH), luteinizing hormone (LH), or estradiol (E_2_) levels as well as other baseline characteristics among groups (*P* > 0.05). The total gonadotrophin (Gn) dosage and duration tended to be less in the GnRH-antagonist group than in the others (*P* < 0.05). No significant differences were found in the implantation rate and clinical pregnancy rate among the groups, but the prolonged GnRH-agonist group showed the highest rates. In addition, no significant differences were present in the number of retrieved oocytes, oocyte fertilization rate, embryo utilization rate, live birth rate, abortion rate, ectopic pregnancy rate, or multiple pregnancy rate in the three groups (*P* > 0.05). Age had a significant effect on both clinical pregnancy and live birth.

**Conclusion:**

For those DOR patients who had undergone ovarian endometriosis cystectomy, the prolonged GnRH-agonist protocol may achieve better clinical IVF-ET outcomes, but there were no significant differences from the other groups. The GnRH-antagonist protocol may reduce the cost and time of drug treatment. Age should be considered for its influence on pregnancy outcome. However, a larger sample size may be needed for further study.

## Background

Endometriosis is a common female disorder that occurs in about 10% of all women of reproductive age and 40–50% of infertile women [1]. Due to the two-child per family policy in China and because more women are postponing childbirth due to pursuit of advanced degrees or developing a professional career, it is likely that infertility related to endometriosis will be encountered more frequently in China. Although endometriosis is a significant reason for progressive pelvic pain and infertility in married women of childbearing age, the etiology and pathogenesis remain unclear. Patients suffering from endometriosis could be adversely affected by distorted tubo-ovarian anatomy [2], triggering inflammation [3, 4] and oxidative damage [5, 6] and resulting in poor quality oocytes [7]. Nearly 17–44% of women with endometriosis have associated endometrioma [8]. Endometriosis involves not only formation of endometriomas, but also endometrial-like lesions in the organs outside of the uterus, for example leading to chocolate cysts or endometriomas inside ovary tissue. The European Society of Human Reproduction and Embryology (ESHRE) updated the guidelines about endometriosis in 2013. Laparoscopic ovarian cystectomy was proposed as the recommended surgical treatment for endometriomas ≥3 cm in diameter [9, 10].

Ovarian reserve is defined as the functional potential of the ovary and reflects the number and quality of the follicles in the ovary [11]. The sensitivity and specificity of ovarian reserve is equivalent to antral follicle count (AFC) and is actually more informative than follicle-stimulating hormone (FSH), estradiol (E_2_), luteinizing hormone (LH), FSH/LH ratio or inhibin-B levels [12]. In recent years, some studies found that laparoscopic cystectomy for ovarian endometriomas may impair ovarian reserve by excessive and accidental removal of healthy ovarian tissue or compromising the ovarian vascular supply and reducing ovarian response to stimulation to some extent [13–18]. Other studies failed to prove its negative influence on ovarian reserve or response [19–21]. Apart from AFC and basal hormone, anti-Müllerian hormone (AMH) level is also demonstrated to predict the magnitude of ovarian reserve as well as controlled ovarian stimulation (COS) responses [18, 22]. Endometrioma cystectomy, especially bilateral surgery, is deleterious with respect to ovarian reserve for AMH, which decreased significantly based on a recent systematic review and meta-analysis and previous studies [12, 18, 23, 24].

Thus, there are conflicting and complex results regarding reproductive outcomes following cystectomy. This is particularly true for women undergoing assisted reproductive technology (ART) because women who underwent the cystectomy may respond less well to gonadotropin (Gn) stimulation and have fewer oocytes retrieved, which results in a higher cancelation rate and lower implantation and pregnancy rates for in vitro fertilization-embryo transfer (IVF-ET). Selecting a proper COS protocol is essential for this population and reproductive clinics should also consider previous surgery experience. Prolonged GnRH-agonist (prolonged GnRH-a) protocol, GnRH-antagonist (GnRH-ant) protocol, and long GnRH-agonist (long GnRH-a) protocol are frequently used in women who underwent IVF treatment. We planned to determine whether these three protocols result in different pregnancy outcomes. In the present study, 342 IVF patients who suffered from diminished ovarian reserve (DOR) induced by endometrioma cystectomy were included and divided into three groups according to the different COS protocols. After IVF, we found that patients treated with the prolonged GnRH-a protocol had a higher implantation rate and clinical pregnancy rate than the GnRH-ant group or the long GnRH-a group.

## Methods

### Population

All women who were referred to the study were selected according to IVF cycles performed in the Department of Human Reproductive Medicine, Beijing Obstetrics and Gynecology Hospital from January 1, 2013 to April 30, 2018. They routinely signed informed consent after a detailed explanation about some subsequent data collection for further study. Inclusion criteria included: age ≤ 40 years; duration of infertility > 12 months; women with a history of a previous laparotomic and/or laparoscopic surgery for unilateral or bilateral ovarian endometrioma(s); and diagnosis of DOR including the following two or more features [25–27]: 10 IU/L ≤ FSH < 25 IU/L or FSH/ LH > 3 on menstrual cycle day 2–3, and AFC ≤5. Patients were excluded if they had a diagnosis of polycystic ovary syndrome (PCOS), uterine fibroids, pelvic tuberculosis, autoimmune diseases, genital organ deformity, metabolic disorders, recurrent miscarriage, recurrent implantation failure, chromosomal abnormality, fertility caused by tubal factor or male factor, a history of other ovarian surgery, or received a steroid or immunosuppressant within the preceding 6 months. The study was approved by the Ethics Committee of the Beijing Obstetrics and Gynecology Hospital, Capital Medical University.

### Study design

This is a retrospective study that assesses the data of 342 patients who received COS in a reproductive center. Excluding intracytoplasmic sperm injection (ICSI), we just included IVF cycles to minimize confounding factors from laboratory operations. These patients were divided into three groups according to different COS protocols: prolonged GnRH-agonist protocol (Group A), GnRH-antagonist protocol (Group B), or long GnRH-agonist protocol (Group C).

For the prolonged GnRH-a protocol (Group A), the following procedure was used. A standard full dose of triptorelin (Diphereline, 3.75 mg, Ipsen Pharma, France; or Decapeotyl, 3.75 mg, Ferring GmbH, Germany) was administered in the early follicular phase for pituitary down-regulation. Down-regulation (no ovarian cysts > 8 mm; E_2_ < 50 pg/ml) was confirmed after 28–42 days. Exogenous Gn (human menopausal gonadotropins, hMG, Livzon Group Livzon Pharmaceutical Factory, China or Highly Purified Menotrophin for Injection, 75 IU, Ferring GmbH, Germany), generally 150 IU to 300 IU/day, was administered until the follicles reached maturity.

For the GnRH-ant protocol (Group B), exogenous Gn (Gonal-F, 75 IU, Merck Serono, Germany; or hMG, 75 IU, Livzon Group Livzon Pharmaceutical Factory, China; or Highly Purified Menotrophin for Injection, 75 IU, Ferring GmbH, Germany), generally 225–300 IU/day, was administered at menstrual day 2–3 until the follicles reached maturity. Daily administration of GnRH-antagonist (Cetrotide, 0.25 mg, QD Merck Serono, Germany) was initiated when Gn was used for six days or when the largest follicle reached a diameter of 13–14 mm.

In the long GnRH-a protocol (Group C), a GnRH-agonist (Decapeotyl, 0.1 mg, Ferring GmbH, Germany) was administered in the luteal phase of the previous cycle. Exogenous Gn (Gonal-F, 75 IU, Merck Serono, Germany; or hMG, 75 IU, Livzon Group Livzon Pharmaceutical Factory, China; or Highly Purified Menotrophin for Injection, 75 IU, Ferring GmbH, Germany) were used at doses ranging between 150 IU/day and 450 IU/day, generally in accordance with age, body mass index (BMI), basal FSH value, size and number of follicles, and E_2_ levels. Patients started serial transvaginal ultrasound from day 5 of ovarian stimulation and serum LH, E_2_, and P measurements until the follicles reached maturity.

When the largest follicle reached 18 mm or at least two follicles reached 17 mm in diameter, human chorionic gonadotropin (hCG) (250 μg, Merck Serono Inc., Geneva, Switzerland) was administered at night. Oocyte retrieval was conducted by transvaginal ultrasound-guided follicular puncture 36 h after hCG trigger. Luteal phase by daily progesterone (Progesterone capsules, 100 mg bid, Xianju Pharma, China and Progesterone soft capsules, 0.2 g tid, Besins Manufacturing, Belgium) was supported from the day of oocyte retrieval. Embryos were graded on day 2/3 based on a ranking system that considered cell number, cell size, fragmentation, and multinucleation. Only one or two high-quality embryos were ultimately chosen to be transferred.

Pregnancies were diagnosed by positive serum hCG 14 days after embryo transfer. A clinical pregnancy was confirmed by visualization of a gestational sac on ultrasonographic examination 28–35 days after the embryo transfer. Luteal support treatment was continued until 10 weeks for confirmed pregnancies.

In the present study, we analyzed the outcomes including number of retrieved oocytes, fertilization rate, clinical pregnancy rate, spontaneous abortion rate, ectopic pregnancy rate, live birth rate, and multiple pregnancy rate in the three groups. Live birth was defined as one or more newborns with vital signs after 28 completed weeks of gestation. Ectopic pregnancy was defined as a pregnancy not occurring inside the uterine cavity. Multiple pregnancy rate was defined as more than one fetus for a pregnancy. To adjust for confounding factors and evaluate baseline characteristics of patients recruited and type of stimulation protocol, we used logistic regression to investigate the potential elements influencing pregnancy and live birth.

### Statistical analysis

Analyses of the data were performed using SPSS (Statistical Package for the Social Sciences) version 23.0 (IBM). Statistically significant differences among non-normally distributed data were determined using non-parametric Kruskal-Wallis H test, Chi-square test or Fisher’s exact test to evaluate continuous or categorical variables and rates as appropriate. Logistic regression was performed for clinical pregnancy and live birth using the following variables: age, BMI, infertility duration, basal hormone levels, antral follicle count, and type of COS. All tests were bilateral and a *P*-value < 0.05 was considered as statistically significant. Measurement results were expressed as medians (lower-upper quartiles) unless stated otherwise.

## Result

### Baseline characteristics of the three groups

During the study period, 342 patients undergoing cystectomy met the criteria and were included in this analysis. The patients’ distribution according to their COS protocol was as follows: group A (113 patients, 33.04%), group B (121 patients, 35.38%), and group C (108 patients, 31.58%). Patient characteristics are summarized in Table 1. The three groups were similar in age, duration of infertility, BMI, basal FSH, basal LH, basal E_2_, and AFC. There were no significant differences among the three groups (*P* > 0.05).

**Table 1:** General characteristics in patients with surgery-induced DOR treated with three controlled ovarian stimulation protocols

	Group A (n = 113)	Group B (n = 121)	Group C (n = 108)	*P* value
Age (years)	33.25 ± 4.05	34.19 ± 3.90	34.12 ± 3.87	NS
Infertility duration (years)	3.89 ± 2.33	4.40 ± 3.07	4.27 ± 2.73	NS
BMI (m/kg^2^)	22.53 ± 3.93	22.84 ± 3.24	22.61 ± 3.46	NS
Type of infertility				NS
Primary infertility	68 (60.18%)	69 (57.02%)	65 (60.19%)	–
Secondary infertility	45 (39.82%)	52 (42.98%)	43 (39.81%)	–
Basal FSH (IU/L)	8.96 ± 3.14	9.30 ± 3.18	8.66 ± 2.50	NS
Basal LH (IU/L)	3.41 ± 2.16	3.69 ± 1.78	3.67 ± 1.99	NS
Basal E_2_ (pg/ml)	52.51 ± 27.28	55.29 ± 32.90	50.20 ± 23.25	NS
Antral follicle counts	4.14 ± 1.43	4.08 ± 1.22	4.41 ± 1.48	NS

Abbreviations: *FSH* follicle-stimulating hormone; *LH* Luteinizing hormone; *E*_*2*_ estradiol;

Statistical differences were calculated using the non-parametric Kruskal-Wallis H test, except for “type of fertilization” where the chi-square test was used

Significant at *P* < 0.05. Values are expressed as mean ± standard deviation unless stated otherwise

### Controlled ovarian stimulation parameters and IVF cycle characteristics

Table 2 summarizes the characteristics of IVF cycles regarding the different COS parameters. Gn stimulation duration was significantly longer in group A (12.90 ± 5.22) compared with group B (9.83 ± 1.74) and group C (10.08 ± 2.22) (*P*<0.05). There was a similar significance tendency in Gn dosage between group A (3493.75 ± 1014.37) and groups B and C [(2581.61 ± 827.11) and (2594.24 ± 1057.56), respectively, *P*<0.05]. Other parameters including hormone levels and thickness of endometrium on hCG day, number of oocytes retrieved, number of fertilized oocytes, and oocytes fertilized rate did not statistically differ among the three groups (*P* > 0.05).

**Table 2:** Controlled ovarian stimulation parameters and IVF cycles characteristics in patients with surgery-induced DOR

	Group A	Group B	Group C	*P* value
Gonadotrophin duration (days)	12.90 ± 5.22^a^	9.83 ± 1.74 ^b^	10.08 ± 2.22 ^b^	<0.001
Gonadotrophin dosage (IU)	3493.75 ± 1014.37^a^	2581.61 ± 827.11^b^	2594.24 ± 1057.56^b^	<0.001
Endometrial thickness on hCG day (mm)	10.89 ± 2.67	10.55 ± 1.94	10.12 ± 2.60	NS
LH on hCG day (IU/L)	2.15 ± 1.60	2.92 ± 2.64	2.60 ± 2.62	NS
E_2_ on hCG day (pg/ml)	1713.34 ± 1167.44	1454.26 ± 853.82	1481.74 ± 916.48	NS
Progesterone on hCG day (ng/ml)	0.97 ± 1.16	0.82 ± 0.73	1.02 ± 1.53	NS
Number of oocytes retrieved	4.03 ± 1.93	3.67 ± 1.92	4.13 ± 2.04	NS
Number of fertilized oocytes	3.11 ± 1.63	2.69 ± 1.52	3.00 ± 1.46	NS
Oocyte fertilization rate (%)	78.29 ± 25.28	73.52 ± 28.92	78.46 ± 24.78	NS

Statistical differences were calculated using the non-parametric Kruskal-Wallis H test, except for “oocyte fertilization rate” where the chi-square test was used

Significant at *P* < 0.05. Values are expressed as mean ± standard deviation unless stated otherwise

^a^Significantly different from group B and group C, *P*<0.001

### Reproductive outcomes of patients after IVF-ET

The reproductive outcomes after IVF-ET treatment are presented in Table 3. The number of transferred embryos was determined in consideration of the conditions of all embryos together with patient preferences. Fresh embryo transfer cycles and total number of transferred embryos were assessed. In Groups A, B, and C, embryo utilization rate (79.49, 78.04, 74.82%, respectively), implantation rate (25.16, 18.01, 17.16%, respectively), clinical pregnancy (45.24, 33.33, 28.99%, respectively), and live birth rate (32.14, 19.54, 24.64%, respectively) were not significantly different (*P* > 0.05). Patients in group A appeared to have higher rates of implantation and pregnancy and were more likely to bear a healthy baby until delivery. The rates of spontaneous abortion (28.95, 34.48, and 15.00%, respectively), ectopic pregnancy (0, 6.90%, and 0, respectively) and multiple pregnancy (5.26%, 0, and 15.00%, respectively) were not statistically different among Groups A, B, and C (*P* > 0.05).

**Table 3:** Reproductive outcomes after IVF in patients with surgery-induced DOR

	Group A	Group B	Group C	*P* value
Total cycles	113	121	108	–
Fresh embryo transfer cycles	84	87	69	–
Fresh transferred embryos	159	161	134	–
Mean number of transferred embryos ddemembyos hello embryos embryos	1.89	1.85	1.94	–
Embryo utilization rate (%, n)	79.49 (248)	78.04 (224)	74.82 (211)	NS
Implantation rate (%, n)	25.16 (40)	18.01 (29)	17.16 (23)	NS
Clinical pregnancy rate (%, n)	45.24 (38)	33.33 (29)	28.99 (20)	NS
Live birth rate (%, n)	32.14 (27)	19.54 (17)	24.64 (17)	NS
Spontaneous abortion rate (%, n)	28.95 (11)	34.48 (10)	15.00 (3)	NS
Ectopic pregnancy rate (%, n)	0	6.90 (2)	0	NS
Multiple pregnancy rate (%, n)	5.26 (2)	0	15.00 (3)	NS

Statistical differences were calculated using the Pearson’s chi-square test or Fisher’s exact test

Significant at *P* < 0.05

### Logistic regression of pregnancy outcomes in patients with surgery-induced DOR

From the logistic regression (Table 4), we observed a significant effect of female age (OR = 0.881, 95% CI (0.799, 0.963), *P* < 0.05) on the clinical pregnancy. For live birth, age also appeared to be a significant variable (OR = 0.845, 95% CI (0.764, 0.934), *P* < 0.05). Other baseline characteristics did not suggest any significant variation in the model.

**Table 4:** Logistic regression of pregnancy outcome in patients with surgery induced DOR

Baseline parameter	B	SE (*b*)	Wald χ^2^	*P* value	ORs	95% CI	
						Lower	Upper
Clinical pregnancy							
Age	−0.127	0.046	7.513	0.006	0.881	0.799	0.963
BMI	0.055	0.050	1.188	0.276	1.056	0.956	1.171
Infertility duration	0.046	0.062	0.551	0.458	1.047	0.934	1.197
Basal FSH	−0.027	0.059	0.213	0.644	0.973	0.857	1.089
Basal LH	−0.018	0.084	0.047	0.828	0.982	0.834	1.169
Basal E_2_	−0.010	0.006	2.611	0.106	0.990	0.977	1.002
AFC	0.139	0.126	1.232	0.267	1.150	0.930	1.546
Live birth							
Age	−0.169	0.051	10.773	0.001	0.845	0.764	0.934
BMI	−0.017	0.049	0.113	0.736	0.984	0.893	1.083
Infertility duration	0.054	0.067	0.655	0.418	1.056	0.926	1.203
Basal FSH	0.064	0.054	1.392	0.238	1.066	0.959	1.186
Basal LH	−0.109	0.093	1.349	0.245	0.897	0.747	1.078
Basal E_2_	−0.007	0.007	1.055	0.304	0.993	0.980	1.006
AFC	0.058	0.128	0.210	0.647	1.060	0.826	1.361

B regression coefficient; SE (*b*) standard errors of regression coefficient; OR odds ratio

The modified Hosmer-Lemshow goodness of fit χ^2^ test statistics were 6.073 (*P* = 0.639) and 2.837 (*P* = 0.944), respectively

## Discussion

Surgical excision of an endometrioma has been the gold standard treatment though there is still a debate about it being detrimental to the ovarian reserve and ultimately associated with DOR [28, 29]. For infertile women after endometrioma cystectomy, spontaneous pregnancy rates may improve, however, IVF is still a primary option for patients still struggling to conceive [30–32]. During our practical clinic work, several protocols chosen for patients with endometrioma cystectomy that induced DOR were individually applied to COS. In addition, patients received the prolonged GnRH-a protocol, GnRH-ant protocol, or long GnRH-a protocol according to personal conditions including menstruation, age, AFC, economic situation, and personal preference. In the present study, we found that clinical pregnancy rate and live birth rate in the prolonged GnRH-a protocol group was higher than the other two groups, but this difference was not significant.

Failed or diminished postsurgical ovarian reserve was reported after undergoing cystectomy for endometrioma [33]. The surgical removal of the cyst might be more catastrophic to certain parts of the vascular system and remaining healthy tissue around the cysts, otherwise an autoimmune reaction caused by a severe local inflammatory response could also harm the ovary function [29]. We observed that every cycle underwent one of the three COS protocols, and had a similar number of oocytes retrieved and oocytes fertilized. In our study, Group A and Group C both used GnRH-agonist, but differed in that patients in Group A usually got at least four-week pituitary down-regulation with one or two triptorelin injections. Over-suppression from long-term administration of the GnRH-agonists may cause poor ovarian response, thus requiring more Gn and taking more time. In Group B, patients endured fewer injections as well as a shorter treatment time, which may be a compromise for families in tough economic conditions. In all cases, the final pregnancy outcomes should be considered.

The embryo utilization rate, implantation rate, clinical pregnancy rate, and live birth rate in patients using prolonged GnRH-agonist protocol were higher compared to the other two protocols. The protocol using long-acting GnRH-agonist may benefit the uterus by contributing to visible or invisible endometriotic lesion atrophy, thus providing an improved internal environment for implantation. Moreover, normal menstrual onset rarely occurs due to the overwhelmingly low level of estrin caused by the inhibition of the hypothalamic pituitary axis, the type of amenorrhea that can increase pinopodes and αvβ3 integrin expression, which represents endometrial receptivity [34–36]. Furthermore, GnRH-agonist, especially for a long period, could evidently lower concentrations of various types of inflammatory cytokines, such as interleukin-1 and tumor necrosis factor, consequently reducing the intra-abdominal toxic effects on oocytes or embryos [37–39].

In our study, we found lower treatment time and pharmaceutical use in the GnRH-antagonist protocol. This protocol has therefore become widespread and is an indispensable part of ovarian stimulation in IVF due to lower risk of ovarian hyperstimulation syndrome (OHSS) and its function as preventing premature luteinization, which is essential for normal follicular development and oocyte maturation [40]. For pituitary desensitization, both daily administration of GnRH-agonist and immediately blocking the secretion of LH with GnRH-antagonist can effectively block premature LH surges. Currently, in view of actual clinical practice, the GnRH-antagonist protocol has been gradually replacing the conventional long GnRH-agonist protocol, which was previously a major choice for all IVF patients.

Abortion rates were not significantly different among the three groups. Data in patients that used GnRH-antagonist protocol gave a higher spontaneous abortion rate but with only a slight significant difference. The same result occurred for ectopic pregnancy rate and multiple pregnancy rate.

Aging was shown to have a negative effect on clinical pregnancy and live birth, as calculated by logistic regression. This finding is in agreement with previous studies showing that advancing age may increase oxidative stress as well as impair ovarian reserve and cytoplasmic quality due in part to decreased androgen levels [41]. Oxidative stress is related to increased granulosa cell apoptosis, which may account for inferior embryo quality and reduced live birth [42]. The condition of oxidative stress may be attributed to poor ovarian function, or have something to do with underlying psychological conditions [43, 44].

We note some deficiencies in the present study. First, its retrospective design inevitably failed to include all relevant data from the patient. Detailed operative recordings, such as time interval between surgery and COS, were not included in the study. Second, the embryo condition and pregnancy outcomes of frozen-thawed embryo transfer (FET) of the included population was lacking, which probably had an influence on overall results. Third, the duration of GnRH-agonist in the prolonged GnRH-agonist protocol lacked subgroups that could be divided based on the specific dose and duration of pituitary suppression. Fourth, we measured the AFC and basal hormone level to roughly define DOR, but postoperative AMH levels were not measured; serum AMH levels may increase with the passage of time after surgery.

## Conclusions

For those IVF-ET patients with DOR induced by ovarian endometrioma cystectomy, prolonged GnRH-a protocol may be an optimal choice, however, the GnRH-ant protocol is less expensive and requires less time for drug treatment. Due to its effect on pregnancy and live birth, age is a significant factor that should not be overlooked in clinical practice. Further studies should use a larger sample size, due to the limited number of cases available, and include all relevant patient data for a comprehensive study.

## Data Availability

All data analyzed during this study are included in this article as tables.

## Abbreviations

AFC: Antral follicle count
AMH: Anti-Müllerian hormone
ART: Assisted reproductive technology
BMI: Body mass index
COS: Controlled ovarian stimulation
DOR: Diminished ovarian reserve
E_2_: Estradiol
ESHRE: European Society of Human Reproduction and Embryology
FET: Frozen-thawed embryo transfer
FSH: Follicle stimulation hormone
Gn: Gonadotrophin
hCG: human chorionic gonadotropin
ICSI: Intracytoplasmic sperm injection
IVF-ET: In vitro fertilization-embryo transfer
LH: Luteinizing hormone
OHSS: Ovarian hyperstimulation syndrome
PCOS: Polycystic ovary syndrome
